# The prevalence of exclusive breastfeeding and its associated factors in Cape Verde

**DOI:** 10.1186/s40795-022-00554-3

**Published:** 2022-08-04

**Authors:** Edna Duarte Lopes, Alzerina Maria Rocha Lima Monteiro, Djenifa Odília Barbosa Fernandes Cardoso Varela, Dulcineia Elisa Lima Rodrigues Trigueiros, Irina Monteiro Spencer Maia, Janice de Jesus Xavier Soares, Nívia Maria da Luz Pires Vieira

**Affiliations:** 1 National Institute of Public Health, Praia, Cape Verde; 2 Praia Health Precinct, Praia, Cape Verde; 3 National Directorate of Health, Praia, Cape Verde

**Keywords:** Exclusive breastfeeding, Breastfeeding, Determinants of breastfeeding

## Abstract

**Background:**

Exclusive breastfeeding (EBF) for the first 6 months of life is essential for maternal and child health. Breast milk is considered the most suitable food for the child in early years of life. Studies carried out in Cape Verde (INE; MSSS, Apresentação dos Principais Resultados Preliminares do IDSR-III, 2018) showed prevalence of EBF below the values recommended by the (WHO & UNICEF, Global Breastfeeding Scorecard, 2018. Enabling Women To Breastfeed Through Better Policies And Programmes, 2019). However, the determinants of EBF across the country have never been identified. The objective of this study is to estimate prevalence and identify the determinants of EBF in Cape Verde.

**Methods:**

This is a quantitative, descriptive and cross-sectional study carried out between July 2018 and March 2019. The study population consisted of 1717 mothers of children aged less than or equal to 2 years, users of the Health Centers of the islands of Santo Antão, S. Vicente, Sal, Santiago and Fogo. A structured questionnaire was applied to mothers through an interview.

The prevalence of exclusive breastfeeding was estimated by frequency analysis. The chi-square test was used to assess the association between the duration of EB and the variables maternity leave, mother’s education, family income and health care variables. In order to identify the determinants of EBF, a binary logistic regression analysis was used.

**Results:**

At the time of data collection, 32.50% of mothers practiced EBF. The present study shows that exclusive breastfeeding is influenced by several factors. The most representatives are maternal age (OR = 0.001*), level of education (OR = 0.028*), parity (OR = 0.004*) and number of prenatal consultations (OR = 0.019*). Receiving breastfeeding counseling was the only health care variable that was associated with the duration of EBF (*p* = 0.029).

**Conclusion:**

In Cape Verde, the prevalence of EBF can be considered reasonable. The present study shows that EBF in the country is influenced by maternal age, level of education, parity and number of prenatal consultations.

**Supplementary Information:**

The online version contains supplementary material available at 10.1186/s40795-022-00554-3.

## Background

Breast milk is the ideal food for children, as it is fully adapted to their needs in the first years of life [1]. Breastfeeding during this period can prevent the onset of several diseases in adult life [2]. The benefits of breastfeeding (BF) for the mother-baby binomial are scientifically recognized due to its nutritional value, immune protection, protection against metabolic diseases [3] and physical and psychological development [2]. Breastfeeding is considered a responsible practice for the prevention of more than 6,000,000 deaths annually in children under 12 months [4].

WHO and UNICEF recommend that children initiate breastfeeding within the first hour of birth and be exclusively breastfed for the first 6 months of life – meaning no other foods or liquids are provided, including water [5].

It is estimated that worldwide, only 41% of children under 6 months are exclusively breastfed, which is below the targets established by the WHO: 50% for 2025 and 70% for 2030 [6].

According to the United Nations (UN) [7] developed countries have the lowest rates of exclusive breastfeeding (23.9%). On the other hand, in less developed countries, the rate of exclusive breastfeeding in the first semester of life is above the global average, reaching 50.8%. The highest rates were found in Rwanda (86.9%), Burundi (82.3%), Sri Lanka (82%), Solomon Islands (76.2%) and Vanuatu (72.6%).

At an economic level, exclusive breastfeeding for at least 6 months would have an impact of 300 billion dollars annually on the world economy [8].

In Cape Verde, according to data from the Continuous Multiobjective Survey [9], the prevalence of exclusive breastfeeding is 30.5%. Provisional data from the third Demographic and Reproductive Health Survey [10] show that the rate of exclusive breastfeeding is approximately 45% of children aged 4 to 6 months. That is, results of studies [9, 10] show that the prevalence of breastfeeding in Cape Verde is lower than the values recommended by the WHO [6]. However, the factors that lead to early weaning are unknown. In this sense, there was a requirement to carry out this study, with the objective of estimating the prevalence and identifying the determinants of EBF in the country. The results are intended to support State’s policies regarding the adequacy of initiatives to promote, protect and support breastfeeding.

## Methods

### Study design

This is a quantitative, descriptive and cross-sectional study carried out in Cape Verde, on the islands with the highest birth rate, between July 2018 and March 2019.

### Subjects

The population for this study consisted of 1765 mothers of children under 24 months who attended the health centers of the selected islands, for immunization and development monitoring of children.

It should be noted that approximately 90% of children aged 6 to 24 months attend health centers for vaccination and development control, in the 2015 statistical report of the Ministry of Health [11].

A probabilistic sample was used, stratified by island and municipality, taking into consideration the number of births per municipality and the number of visits to health centres for children under 24 months of age in 2015, according to the Ministry of Health Statistics Report, referring to 2015 (MSSS, 2017). The islands with the greatest number of births in 2015 were chosen. After the exclusion of all incomplete questionnaires, the population consisted of 1717 individuals. Criteria for inclusion were: to have been a mother for less than 24 months, be present at the Health Centre on the days set aside for data collection and voluntarily agree to participate in the project. The exclusion criterion is being a carrier of a clinical condition and mental retardation or disturbance that prevented the response to the questionnaire. All incomplete questionnaire are deleted.

### Instruments

A structured questionnaire, adapted by the researchers from a model used in a Brazilian study, consisting of 39 items, was used to collect demographic, socioeconomic data, information on pre and postnatal care, mother’s health status, child birth and on the determinants of breastfeeding.

### Data collection

The data was collected on the islands of Santo Antão, São Vicente, Sal, Santiago and Fogo, from July 2018 to March 2019.

A structured questionnaire was applied to the mothers through interviews, after consultations to control the children’s development, in the health centers of the 5 selected islands.

The teams of surveyors, made up of two health technicians (nutritionists, nurses or biomedical engineers) from each selected municipality, were trained to collect data on February 7, 2018. The technicians received training in different areas: i) basics notions of breastfeeding; ii) methods of approaching the respondents and, iii) application of questionnaires. They also received a support manual, containing all the guidelines regarding the collection of study data.

A pre-test was conducted, with the application of the questionnaire to a group of 30 mothers of children under 24 months of age to assess the clarity of the language and the ease of completing the questionnaire. These questionnaires were not considered in the study.

Each field team was supported by a supervisor (nutritionist or psychologist).

### Statistical analysis

Data were analyzed using the Statistical Package for the Social Sciences software (SPSS, v. 26).

In the analysis of the reasons that lead mothers to start and stop breastfeeding, and the prevalence of exclusive breastfeeding in the first 6 months of life, proportions were used.

The association between the duration of exclusive breastfeeding and the variables maternity leave, mother’s education, family income, and health care variables (number of prenatal consultations, prenatal care location, child’s birthplace, counseling on breastfeeding, breastfeeding in the first hour after delivery and child’s birth weight) was performed using Chi-Square (χ2) test.

A binary logistic regression analysis was used to determine the influence of the variables maternal age, level of education, marital status, family income, area of residence, pregnancy planning, prenatal care, number of prenatal consultations, maternity leave, weight of baby at birth, breastfeeding in the first hour after birth, parity and breastfeeding counseling.

Statistical analysis was performed at a significance level of 0.05.

### Ethical aspects

The research was conducted according to the ethical standards and guidelines of Decree-Law No. 26/2007, dated 30 July, was approved by Deliberation No. 42/2017 by the National Ethics Committee on Health Research (Portuguese acronym of CNEPS).

Before the application of the questionnaire, the mothers were informed about the objectives, the importance of carrying out the investigation and the methodological procedures.

All mothers agreed to participate in the research, by signing an informed consent form, according to the ethical principles recommended in the country.

## Results

### Characteristics of study participants

The sample consisted of 1717 subjects attended at Health Centers in 11 municipalities in the country, aged between 13 and 48 years (Md = 26 years). Most were single (58.9%), had high school (57%), declared a monthly family income less than 30.000$00 (71.1%), lived in urban areas (70.5%) and reported having more than one child (58.3%) (Table 1).

**Table 1:** Background characteristics of participants

Variables	N	%
**Age**		
13–21	461	26.8
22–26	461	26.8
27–31	409	23.8
32–48	386	22.5
**Marital status**		
Single	1008	58.9
Married/couple life	697	40.8
Separated/Divorced	5	0.3
**Education**		
Illiteracy	17	1
Basic education	428	25
High school	978	57
Professional qualification	221	12.9
Bachelors’s degree	71	4.1
**Employment**		
Unemployed	1018	59.3
Employed	699	40.7
**Family income**		
< 15,000$00	678	39.5
15.000$00 a 29.000$00	542	31.6
30.000$00 a 60.000$00	256	14.9
>60.000$00	177	10.3
Didn’t know/didn’t want to answer	64	3.7
**Residence**		
Urban	1210	70.5
Rural	506	29.5
**Island of residence**		
Santo Antão (16.8%)		
Ribeira Grande	200	11.6
Paul	54	3.2
Porto Novo	35	2.0
S. Vicente (17.7%)	304	17.7
Sal (15.3%)	263	15.3
Santiago (35.7%)		
Santa Catarina	272	15.8
Praia	331	19.3
Rª Grande de Santiago	9	0.5
Fogo (14.5%)		
São Filipe	83	7.9
Mosteiros	135	4.8
Sta. Catarina do Fogo	31	1.8
**Parity**		
1 child	716	41.7
2 children	522	30.4
3 children	278	16.2
>3 children	201	11.7

### Prevalence of exclusive breastfeeding

The prevalence of exclusive breastfeeding was 32.5% (Table 2).

**Table 2:** Prevalence of exclusive breastfeeding

	n	%
**Mothers who practiced EBF**	558	32.5
**Mothers who did not practice EBF**	1159	67.5
**Total**	1717	100

### Reasons for adherence to exclusive breastfeeding

Most mothers reported that the last child had 6 months or more (74.4%). It was found that most mothers were aware of the benefits of breastfeeding for their child in the first 6 months of life (Table 3).

**Table 3:** Reasons for adherence to exclusive breastfeeding

Answers	N	%
**Breast milk is the best food for the baby**	319	18.7
**Breast milk is a healthy diet**	153	8.9
**Breast milk helps child’s development**	90	5.2
**I was advised**	73	4.3
**Breast milk prevents child from infections**	55	3.2
**I had enough milk**	51	3.0
**It’s a child’s right**	45	2.6
**It’s an act of love/bonding**	27	1.6
**It’s more practical**	12	0.7
**Breastfeeding protects the mother’s health**	10	0.6
**It is the mother’s duty/pleasure**	10	0.6
**Breast milk is cheaper**	8	0.5
**Breast milk protects the baby from allergies**	1	0.1
**The child needs breastfeeding**	1	0.1
**More than an option**	817	47.9
**All previous answers**	13	0.8
**Did not know**	14	0.8
**Did not specify**	6	0.5
**Total**	1705	100

### Reasons for non-adherence to exclusive breastfeeding

Having to work was the main reason for mothers’ non-adherence to breastfeeding, followed by the perception that the amount of milk was insufficient (Table 4).

**Table 4:** Reasons for non-adherence to exclusive breastfeeding

Answers	N	%
**The mother had to work/study**	68	20.9
**The mother felt that the milk was insufficient**	52	16
**The mother did not liked/wanted to breastfeed**	50	15.4
**The child did not accept**	27	8.3
**Medical advice**	20	6.1
**Breast problems**	9	2.8
**The mother thought her milk was weak**	8	2.5
**Aesthetics**	7	2.2
**Pregnancy**	5	1.5
**Child/mother trip**	5	1.5
**Use of bottle and pacifiers**	2	0.6
**Child’s illness**	4	1.2
**More than one option**	37	11.4
**Started to feed child with water/food**	31	9.6
**Total**	325	100

### Associated factors of exclusive breastfeeding

The result of the binary logistic regression analysis indicates that, in general, ages below 26 years (OR = 2.10), secondary or higher education levels (OR = 1.55), more than 8 prenatal visits (OR = 1.61) and having more than 2 children (OR = 1.88) are significantly associated with breastfeeding (*p*<0.05) (Table 5).

**Table 5:** Associated factors of exclusive breastfeeding

Variables	OR	OR (95% CI)	***p***-value
**Maternal age (years)**			
< 26 years old	2.10	(1.362–3.246)	0.001*
≥ 26 years old	1.00	–	–
**Education**			
High school and Bachelors’s degree	1.55	(1.050–2.292)	0.028*
Up to elementary education	1.00	–	–
**Marital status**			
Single	1.27	(0.876–1.829)	0.209
Married/cohabiting	1.00	–	–
**Family income**			
CVE < 15,000	1.01	(0.682–1.504)	0.949
CVE ≥ 15,000	1.00	–	–
**Location of residence**			
Rural	1.07	(0.715–1.591)	0.754
Urban	1.00	–	–
**Pregnancy planning**			
Yes	1.06	(0.726–1.544)	0.768
No	1.00	–	–
**Prenatal care**			
Yes	1.46	(9.487–4.402)	0.498
No	1.00	–	–
**N of prenatal visits**			
≥ 8 visits	1.61	(1.082–2.392)	0.019*
< 8 visits	1.00	–	–
**Maternity leave**			
Yes	1.03	(0.411–2.561)	0.956
No	1.00	–	–
**Child’s birth weight**			
Low	1.20	(0.536–2.682)	0.659
Normal	1.00	–	–
**Breastfeeding in the 1st hour after delivery**			
Yes	1.07	(0.680–1.689)	0.766
No	1.00	–	–
**Parity**			
More than 2 children	1.88	(1.225–2.883)	0.004*
Up to 2 children	1.00	–	–

*p < 0.05

There was a positive association between exclusive breastfeeding and the variable “counseling on breastfeeding” (χ^2^_(1717;2)_ = 4.926; *p* = 0.029 < *p* = 0.05) (Additional file 1: Appendix I). However, there was no association between maternity leave and exclusive breastfeeding (χ^2^_(1059;1)_ = 0.032; *p* = 0.865) (Additional file 2: Appendix II).

## Discussion

This study estimated the prevalence of EBF, as well as their associated factor in Cape Verde. The World Health Organization has recommended exclusive breastfeeding for 6 months as a way to prevent morbidity among children [12].

The WHO has set the goal of achieving 50% exclusive breastfeeding worldwide by 2025 [13]. However, in 2017 the percentage of EBF was situated at 41% [13]. The prevalence of exclusive breastfeeding in Cape Verde was 32.5%, which can be considered reasonable. This result is greater than that of two studies carried out in Brazil [14, 15].

Mothers emphasized that the main reasons to begin breastfeeding, the fact that breast milk is “the best food for the baby”, “a healthy food”, as well as its importance for “child development”, concur with the findings of other studies [16, 17].

On the other hand, mothers indicated as relevant causes of early weaning, “having to work or study”, “insufficient milk” and “mothers’ refusal to breastfeed”, corroborating other research findings [18–22]. The results obtained also corroborate those of studies conducted in Brazil [14, 23], concerning the lack of willpower to breastfeed, the lack of time due to work/study, rejection of the baby and poor milk production, albeit in very different proportions.

In this study, 81% of mothers correctly identified the period recommended by the World Health Organization (WHO) for exclusive breastfeeding, corroborating several studies [17, 22, 24, 25]. However, this knowledge was not enough to guarantee exclusive breastfeeding [17], according to this author, besides knowledge and attitudes, there is a need to consider culture and traditions.

Maternity leave facilitates exclusive breastfeeding [26]. In a survey conducted by Ortelan (2019), maternity leave of at least 6 months was considered to be associated with an 8.9% increase in exclusive breastfeeding [20]. Recent studies [1, 20, 21, 25–27] show that the rate of breastfeeding and EBF declines when the mother goes back to work. The prevalence of exclusive breastfeeding is higher for mothers on maternity leave compared with unemployed mothers [26, 28, 29], which demonstrates the facilitative effect of maternity leave versus EBF. However, this study does not support the results of the mentioned studies. In fact, maternity leave did not significantly affect breastfeeding or exclusive breastfeeding.

In the current study, only the variables of maternal age, maternal education, number of prenatal visits and parity showed significant correlation with breastfeeding. Mothers under the age of 26 appear to be more likely (OR = 0.001) to breastfeed than mothers over the age of 26, which are inconsistent with recent research findings [14, 25, 28–33].

Mothers with a secondary level or bachelors’s degree are more likely (OR = 1.55) to breastfeed when compared to mothers with an education level up to elementary school, which corroborates the results of a study carried out in Sub-Saharian Africa [29] and Brazil [34], according to which the duration of breastfeeding is longer in mothers with higher education, and also with another study, where the prevalence of exclusive breastfeeding was lower among mothers who had not completed high school and whose income was below the minimum wage [3, 29, 35]. Many studies reported a significant and positive association between counselling and EBF. In the present study, mothers who had more than 8 prenatal medical appointments were more likely (OR = 1.88) to breastfeed more, which is in agreement with the results of other studies [29, 35, 36].

It was noted that mothers with more than two children were more susceptible (OR = 1.88) to breastfeeding than mothers with up to two children, confirming results from other studies [34, 36].

In this study, educational and purchasing power variables were not statistically significant for exclusive breastfeeding. Similar results were observed in a number of studies [15, 37, 38].

Among the variables that include health care in the prenatal period, only number of prenatal visits greater than or equal to 8 (*p* = 0.019) and counseling on breastfeeding (*p* = 0.029) are associated with exclusive breastfeeding, which supports the findings of the study by Alves et al. [3, 29, 35] and objected to the conclusions drawn by Ferreira et al. [37].

## Conclusion

In Cape Verde, the prevalence of exclusive breastfeeding may be considered reasonable. Mothers aged 26 or younger, who had high school and higher education, had more than two children and attended eight or more prenatal medical appointments, were more compliant with exclusive breastfeeding. The perception that breastmilk is the “best food for the baby” is the main reason for adhering to EBF. However, the need to work or study has been identified as the major reason for not adhering to the EBF. Among the variables in health care, breastfeeding counselling appears to be the only positive influence on EBF. The findings of the study must be interpreted in light of the limitations presented.

### Limits and recommendations for further studies

The following points should be considered in future research:

- Use of a sample stratified by island and county throughout the country, representing mothers with children aged or 6 months, instead of those seeking health care;
- Verification of the reasons for mothers not completing the number of prenatal visits stipulated by the WHO;
- Analysis of the gap between pre-natal care settings/areas.

## Supplementary Information

**Additional file 1: Appendix 1.**

**Additional file 2: Appendix 2.**

## Data Availability

The datasets generated and/or analyzed in this study are available from the corresponding author (EDL) upon request with reasonable justification and are stored at the National Institute of Public Health in a repository with limited and controlled access. The data are not publicly available because they contain confidential information that may compromise the privacy/consent of the participants.

## Abbreviations

BF: Breastfeeding
EBF: Exclusive breastfeeding
WHO: World Health Organization
UN: United Nations
