# Supplementary material for: The prevalence of exclusive breastfeeding and its associated factors in Cape Verde
Source: BMC Nutr. 2022 Aug 4;8:74. doi: 10.1186/s40795-022-00554-3 (PMC9351167; doi:10.1186/s40795-022-00554-3)
Supplement: Supplementary file 1 — Additional file 1: Appendix 1. [file 40795_2022_554_MOESM1_ESM.doc]

Appendix I. Exclusive breastfeeding gradient estimative to health care variation

| **Variables** | **EBF** | |  |  |
| --- | --- | --- | --- | --- |
| **Number of prenatal visits** | **Sim n(%)** | **Não n(%)** | **χ^2^** | **P-valuer** |
| **˂ 8** | 315 (56.5) | 620 (53.5) |  |  |
| **≥ 8** | 220 (39.4) | 495 (42.7) | 1.682 | 0.431 |
| **Did not remember** | 23 (4.1) | 44 (3.8) |  |  |
| **Prenatal follow-up** | **Sim n(%)** | **Não n(%)** | **χ^2^** | **P-valuer** |
| **Health Center** | 458 (84.2) | 934 (83.2) |  |  |
| **Hospital** | 22 (4.0) | 55 (4.9) | 3.501 | 0.321 |
| **Private clinic** | 30 (5.5) | 46 (4.1) |  |  |
| **Hospital and clinic** | 34 (6.3) | 88 (7.8) |  |  |
| **Last child's birthplace** | **Sim n(%)** | **Não n(%)** | **χ^2^** | **P-valuer** |
| **Hospital** | 525 (94.9) | 1098 (95.9) |  |  |
| **Health Center** | 17 (3.1) | 34 (3.0) | 2.542 | 0.468 |
| **At home** | 9 (1.6) | 9 (0.8) |  |  |
| **Other** | 2 (0.4) | 4 (0.3) |  |  |
| **Breastfeeding counseling** | **Sim n(%)** | **Não n(%)** | **χ^2^** | **P-valuer** |
| **Yes** | 508 (91) | 1013 (87.4) |  |  |
| **No** | 50 (9) | 146 (12.6) | **4.926** | **0.029*** |
| **Breastfeeding in the first hour after delivery** | **Yes n(%)** | **No n(%)** | **χ^2^** | **P-valuer** |
| **Yes** | 444 (80.1) | 915 (79.6) |  |  |
| **No** | 110 (19.9) | 235 (20.4) | 0.078 | 0.797 |
| **Child birth weight** | **Sim n(%)** | **Não n(%)** | **χ^2^** | **P-valuer** |
| **Low weight** | 526 (94.3) | 1092 (94.9) |  |  |
| **Normal** | 32 (5.7) | 59 (5.1) | 0.276 | 0.646 |

*p ˂ 0.05
