# Supplementary material for: The prevalence of exclusive breastfeeding and its associated factors in Cape Verde
Source: BMC Nutr. 2022 Aug 4;8:74. doi: 10.1186/s40795-022-00554-3 (PMC9351167; doi:10.1186/s40795-022-00554-3)
Supplement: Supplementary file 2 — Additional file 2: Appendix 2. [file 40795_2022_554_MOESM2_ESM.docx]

Appendix II. Relationship between maternity leave and prevalence of exclusive breastfeeding

| Maternity leave | EBF | | χ^2^ | P-valuer |
| --- | --- | --- | --- | --- |
|  | **Yes**  **n (%)** | **No**  **n (%)** |  |  |
| Work outside with maternity leave | 14 (3.8%) | 25 (3.6%) | **0.032** | 0.865 |
| Work outside without maternity leave | 352 (96.2%) | 668 (96.4%) |  |  |

*p ˂ 0.05
